# An Update on the Multifaceted Roles of STAT3 in the Heart

**DOI:** 10.3389/fcvm.2019.00150

**Published:** 2019-10-25

**Authors:** Zeina Harhous, George W. Booz, Michel Ovize, Gabriel Bidaux, Mazen Kurdi

**Affiliations:** ^1^Laboratory of Experimental and Clinical Pharmacology, Faculty of Sciences, Doctoral School of Sciences and Technology, Lebanese University, Beirut, Lebanon; ^2^Univ-Lyon, CarMeN Laboratory, INSERM 1060, INRA 1397, University Claude Bernard Lyon1, INSA Lyon, Oullins, France; ^3^IHU OPeRa, Groupement Hospitalier EST, Bron, France; ^4^Department of Pharmacology and Toxicology, School of Medicine, The University of Mississippi Medical Center, Jackson, MS, United States

**Keywords:** STAT3, cardioprotection, cardiac pathologies, myocardial infarction, genomic functions

## Abstract

Signal transducer and activator of transcription 3 (STAT3) is a signaling molecule and transcription factor that plays important protective roles in the heart. The protection mediated by STAT3 is attributed to its genomic actions as a transcription factor and other non-genomic roles targeting mitochondrial function and autophagy. As a transcription factor, STAT3 upregulates genes that are anti-oxidative, anti-apoptotic, and pro-angiogenic, but suppresses anti-inflammatory and anti-fibrotic genes. Its suppressive effects on gene expression are achieved through competing with other transcription factors or cofactors. STAT3 is also linked to the modification of mRNA expression profiles in cardiac cells by inhibiting or inducing miRNA. In addition to these genomic roles, STAT3 is suggested to function protectively in mitochondria, where it regulates ROS production, in part by regulating the activities of the electron transport chain complexes, although our recent evidence calls this role into question. Nonetheless, STAT3 is a key player known to be activated in the cardioprotective ischemic conditioning protocols. Through these varied roles, STAT3 participates in various mechanisms that contribute to cardioprotection against different heart pathologies, including myocardial infarction, hypertrophy, diabetic cardiomyopathy, and peripartum cardiomyopathy. Understanding how STAT3 is involved in the protective mechanisms against these different cardiac pathologies could lead to novel therapeutic strategies to treat them.

## Introduction

Cardiovascular diseases are among the top challenges to global health, constituting a leading cause for morbidity and mortality worldwide. According to the World Health Organization (WHO), these diseases result in 17.5 million deaths globally per year (1). This alarming situation increasingly excites research in the cardiovascular field, aiming to understand the mechanisms underlying different cardiac pathologies. This represents a prerequisite for developing and setting therapeutic cardioprotective strategies. A remarkable body of evidence has shown that STAT3 plays an important role in cardioprotection, rendering it a prominent focus of interest. It participates in mechanisms that contribute to protection against myocardial infarction, fibrosis, hypertrophy, hypertension, myocarditis, diabetic cardiomyopathy, and peripartum cardiomyopathy. Several previous reviews have generally addressed STAT3 functioning at the cellular level in the heart, focusing on its signaling, activation, and post-translational modifications; other reviews have focused solely on a particular cardiac pathology (1, 2). This review presents a critical appraisal of the physiological and pathological roles of STAT3 in the heart. We focus on the involvement of STAT3 in cardiac myocyte and heart pathologies, starting from a general overview of STAT3 and addressing its activation, post-translational modifications, and different cellular pools.

## STAT3 Overview

### Structure

STAT3 is an 89 kDa protein encoded by the *STAT3* gene. It is 770 amino acids in length and is composed of six functional domains. The NH_2_-terminal domain participates in higher order complex formation. This is followed by the coiled-coil domain involved in protein-protein interactions with co-regulators and transcription factors. Next is the DNA binding domain with a sequence specificity for an interferon gamma (INFγ) activated sequence (GAS) present in the promoter region of specific genes. Following that is the linker domain located directly before the Src homology-2 (SH2) domain, which mediates receptor recruitment and STAT3 dimerization *via* intermolecular phosphorylated tyrosine-SH2 interactions. The transcription activation domain (TAD), located in the COOH-terminal region, is where regulatory phosphorylation occurs (3–5).

STAT3 exists as two main isoforms, STAT3α (generally referred to as STAT3) and STAT3β. Compared to STAT3α, STAT3β is the truncated form lacking the 55-residue C-terminal transactivation domain. This splice variant can be phosphorylated at the tyrosine 705 (Y705) residue, but unlike STAT3α lacks serine 727 (S727). STAT3β can still bind to DNA as a homo- or heterodimer (with STAT3α or other transcription factors) (3, 6). The role of STAT3β in the heart is unclear.

The main function of STAT3 is the regulation of gene transcription. STAT3 is modified at specific residues by a number of post-translational modifications with functional consequences, mainly acetylation and phosphorylation. In addition, methylation, ubiquitination, s-nitrosylation, and glutathionylation occur (5). In addition, recent evidence was reported that S-palmitoylation of STAT3 promotes its dimerization and transcriptional activation (7).

### Post-translational Modifications

#### Phosphorylation

##### Tyrosine phosphorylation

STAT3 is activated through phosphorylation of its tyrosine 705 residue (Y705) by receptor and non-receptor tyrosine kinases. This activation facilitates STAT3's homo/hetero-dimerization and DNA binding (5, 6). Tyrosine phosphorylation occurs in response to the stimulation by a variety of cytokines, such as IL-6, interferon gamma (IFNγ), and erythropoietin, in addition to growth factors such as epidermal growth factor (EGF) and fibroblast growth factor (FGF) (8–12). Tyrosine phosphorylated STAT3 molecules form dimers that translocate from the cytoplasm to the nucleus and bind to DNA. As a result, STAT3 regulates expression of genes that control cell proliferation, differentiation, and apoptosis.

In cardiomyocytes, agonists with pathophysiological importance engage membrane receptors coupled to the activation of JAK1 and JAK2 tyrosine kinases, and consequently lead to STAT3 tyrosine phosphorylation. These agonists include, in addition to IL-6 and leukemia inhibitory factor (LIF), tumor necrosis factor alpha (TNFα), IFNγ, erythropoietin, and granulocyte-colony stimulating factor (G-CSF). The Src family kinases and bone marrow kinase on chromosome X (BMX) also induce tyrosine phosphorylation of STAT3 in cardiac cells (5).

##### Serine phosphorylation

Serine/threonine kinases have been shown to phosphorylate STAT3 on S727 in the cytoplasm or nucleus. The involvement of serine phosphorylation in STAT3 activities was first reported in 1995 by Zhang et al. who studied the importance of such phosphorylation for DNA binding (13). S727 was later demonstrated through mutational studies to be required for maximal transcriptional activity, but not for DNA binding (14, 15). Serine phosphorylation may boost the transcriptional activity of STAT3 by recruiting transcriptional co-factors, such as histone acetyltransferase p300/CBP (13, 16). However, there are reports as well that it may act to decrease tyrosine phosphorylation (17, 18).

Multiple kinases are responsible for S727 phosphorylation, depending on the nature of the activating signal. Mitogen activated protein kinase (MAPK) family members are mainly reported to induce this phosphorylation in response to different extracellular stimuli. The MAPK family is composed of extracellular signal-regulated kinase (ERK), induced by growth factors and cytokines, as well as JNK/stress-activated protein kinase and p38/HOG1 (p38 MAPK), both activated by pro-inflammatory cytokines and environmental stresses (19, 20). Among them, the best characterized serine kinases inducing S727 phosphorylation are ERK1/2. It is important to note that JNK and p38 are considered mediators of ischemia-reperfusion injury, where they upregulate inflammatory cytokines and activate mitochondria-dependent death pathways (21). JNK and p38 MAPKs are activated by TNFα, which is activated during cardiac conditioning (22). During heart failure, the expression of the heat shock protein H11 kinase/Hsp22 increases as a stress-response mechanism. This was shown to increase the phosphorylation of STAT3 on S727, along with an increase of mitochondrial respiration ensuring cardioprotection (23).

#### Acetylation

Besides phosphorylation, the function of STAT3 is also regulated by p300-mediated acetylation of lysine residues within its NH2 terminus (K49, K87) and SH2 (K685) domains. Acetylation of K49 and K87 was shown to be important for transcription through strengthening or enhancing the interaction of STAT3 with the histone acetyltransferase p300, which stabilizes the enhanceosome assembly and ensures retention of STAT3 in the nucleus (24, 25). In addition, by recruiting p300, STAT3 positively affects the accessibility of other transcription factors to some promoters. K685 acetylation is important for STAT3 dimer formation and transcription enhancement at certain genes, perhaps independent of tyrosine phosphorylation (16, 26, 27). In addition, evidence was reported that increased p300 acetyl transferase activity in neonatal rat ventricular myocytes positively affected STAT3 activation and half-life (28).

#### S-Nitrosylation

It has become clear that nitric oxide (NO) is an endogenous regulator in cardiovascular physiology and cellular respiration. The presence of NO provokes S-nitrosylation, a ubiquitous post-translational modification, by selective addition of nitric oxide (NO) to specific cysteine residue(s) of proteins forming S-nitrosothiol (SNO). This redox-related post-translational modification can inhibit STAT3 activation. STAT3 was observed to be S-nitrosylated on C259, which inhibited JAK2-mediated Y705 phosphorylation. For instance, endogenous NO from iNOS or treatment with s-nitrosoglutathione inhibited STAT3-induced gene expression and proliferation in microglial cells. It has not been reported yet if STAT3 is s-nitrosylated in the heart under conditions where iNOS is upregulated, such as the ischemic and failing myocardium (29–31).

#### S-Sulfhydration

*S*-sulfhydration is a post-translational modification involved in many physiological and pathological processes. S-sulfhydration yields hydropersulfide (–SSH) in the active cysteine residue of a protein, which mediates most of the cellular functions induced by hydrogen sulfide (H_2_S). Some preclinical studies have reported that the administration of hydrogen sulfide (H_2_S) decreases myocardial injury, protects blood vessels, limits inflammation and regulates blood pressure. In the context of STAT3, Wu et al. reported that *S*-propargyl-cysteine (SPRC), a producing agent of endogenous hydrogen sulfide (H_2_S), possesses cardioprotective efficacy through the activation of STAT3 phosphorylation downstream of gp130. In another study, Xin et al. reported that H_2_S ameliorated IL-6-induced STAT3 acetylation, which was accompanied by a decreased inflammatory response in patients with anemia. Although the presence of H_2_S provokes sulfhydration, it seems that there is no direct sulfhydration of STAT3 reported in the presence of H_2_S, but rather the presence H_2_S induces phosphorylation or acetylation (31–34).

## STAT3 Pools and Translocation

### Nuclear STAT3

STAT3 is predominately cytoplasmic in resting cells, but its role as a DNA-binding transcription factor naturally depends on its ability to gain entrance to the nucleus. This facilitates its ability to transduce signals from the receptor to the appropriate target genes in the nucleus (35, 36). At the genomic level, STAT3 positively regulates the expression of anti-apoptotic (Bcl-2 and Bcl-xl) (6) and anti-oxidative (MnSOD and Metallothionein MT1/MT2) proteins (37). It reduces the expression of the pro-apoptotic factor BAD and regulates expression of some growth factors, such as vascular endothelial growth factor (VEGF) (38). The deletion of STAT3 in cardiac myocytes eliminated the upregulation of the anti-apoptotic (Mcl-1, Bcl-xL, c-FLIP) and cardioprotective (COX-2 and HO-1) proteins, which reflects the cardioprotective roles of STAT3 as a transcription factor (39).

### Mitochondrial STAT3

Although it was originally assumed that STAT3-dependent biological effects are due to its potency as a transcription factor capable of regulating gene expression, evidence of its mitochondrial presence and activity has emerged (40, 41). In this context, STAT3 has been reported to control electron transport chain (ETC) activity and adenosine 5′-triphosphate (ATP) production (37, 42–46). This activity has been suggested to be dependent on S727 phosphorylation in different types of cells including cardiomyocytes (46, 47). Among the approaches used for studying STAT3's mitochondrial activity are STAT3 KO models, pharmacologic inhibition, and the overexpression of a mitochondria-targeted STAT3 with mutated DNA-binding domain (MLS-STAT3E) (37, 46, 48). However, these approaches may be questioned for several reasons. First, STAT3 is known to regulate genes involved in metabolism and mitochondrial activity (40), so the use of KO STAT3 models may be misleading in the context of a mitochondrial presence for STAT3. Second, the use of MLS-STAT3E is non-physiological, since it provokes forced STAT3 mitochondrial translocation and may point toward a misleading involvement for STAT3 in the regulation of mitochondrial activity. Therefore, the mitochondrial role has sparked controversy and seems unclearly defined in cardiac cells specifically. In this regard, Harhous et al. recently reported that STAT3 is not detected in mitochondria of adult cardiac myocytes from C57bl5/J mice and does not regulate mitochondrial calcium retention capacity or respiration activity (49). Similarly, another previous study performed on mouse heart mitochondria also failed to detect pS727-STAT3 in mitochondria (50). Overall, the presence of STAT3 in mitochondria of cardiac cells remains a controversial issue.

## Roles of STAT3 in Cardiomyocyte Pathologies

### STAT3 in Ischemia-Reperfusion Injury

Ischemia-reperfusion (IR) injury is a pathological state caused by an initial low supply of blood to a specific area, followed by the restoration of perfusion and reoxygenation (reperfusion). This injury is associated with a profound inflammatory response (51, 52). During ischemia, apoptotic and necrotic cell death occur. However, cell death is enhanced during reperfusion, and thus contributes mainly to IR injury (53, 54). A significant body of *in vivo* and *in vitro* evidence suggests that STAT3 activation results in a cardioprotective response to ischemia and IR injury.

#### STAT3 Phosphorylation During IR Injury

During ischemia, STAT3 is generally phosphorylated on its Y705 residue with a further increase in phosphorylation of this residue during reperfusion (1, 55–61). However, phosphorylation of S727 was reported to be suppressed in the nucleus and mitochondria during reperfusion (1). The pattern of STAT3 activation has been described by Somers et al. who reported that pY705-STAT3 was increased in mitochondria, but decreased in the cytosol after a short reperfusion period (62). In the context of IR injury, Meier et al. recently revealed that oxidative stress and cytokines, which are supposedly important mediators of IR injury, dynamically regulated mitochondrial STAT3. Their results highlighted that H_2_O_2_ and different cytokines induced a rapid loss of STAT3 (within few minutes) from mitochondria, and a rapid recovery also, dependent on the phosphorylation of the S727 residue. Although this study was performed on different cell types, it was not assessed in cardiac cells (63).

#### Upstream Activators of STAT3 During IR Injury

A notable relationship exists between cardiac IR injury and the production of IL-6 cytokines, which are among the most prominent activators of STAT3 (19, 60, 64–66). These cytokines were shown to mediate cardioprotection against acute MI through JAK-STAT3 activation (60, 61). In addition to cytokines, angiotensin II has been linked to STAT3 activation during ischemia. In this regard, Omura et al. reported that angiotensin II mediated the activation of myocardial JAK-STAT3 in acute myocardial ischemia in an *in vivo* study with Wistar rats (59). Moreover, insulin was shown to induce cardioprotection against IR injury through activation of the JAK-STAT3 pathway (61). A number of other factors have been demonstrated to activate STAT3 in the context of IR injury with a protective effect observed, including TNF-α, sphingosine-1-phosphate, HDL, ethanolamine, and melatonin (1).

#### Mechanisms Contributing to the Protection Against IR Injury

Activated STAT3 contributes to protection against IR injury through decreasing ROS generation and apoptosis, delaying MPTP opening, and increasing angiogenesis.

STAT3 upregulates anti-apoptotic proteins. Knockout of STAT3 induced a significant increase in infarct size associated with enhanced apoptosis of cardiomyocytes following IR (1, 57). Using a cardiomyocyte-specific STAT3 KO model, Bolli et al. reported that STAT3 deletion eliminated delayed preconditioning-induced upregulation of anti-apoptotic (Bcl-xL, c-FLIPL, Mcl-1, and c-FLIPS) and cardioprotective (HO-1 and COX-2) proteins (39). Using a similar model, Enomoto et al. reported that STAT3 expression in cardiac myocytes contributes to remodeling during the subacute phase of MI (67). Moreover, inhibition of the JAK/STAT pathway by AG490 significantly inhibited STAT3 phosphorylation in rat hearts and increased caspase-3 activity and Bax expression in viable myocardium following infarction (56).

Concerning ROS regulation, STAT3 is capable of decreasing ROS in cardiomyocytes indirectly through upregulating the expression of antioxidants and (purportedly) directly by decreasing ROS generation in mitochondria. On the one hand, STAT3 upregulates expression of ROS scavengers and anti-oxidants: metallothioneins and MnSOD (68, 69). On the other hand, STAT3 decreases ROS generation by complexes I and III of the ETC. Boengler et al. reported that the STAT3 inhibitor Stattic decreased ADP-stimulated mitochondrial respiration and enhanced calcium-induced opening of the mitochondrial permeability transition pore (MPTP) as a result of ROS generation (70), although others reported contrary findings (49). In addition to these roles, other cardioprotective roles of STAT3 have been ascribed to the involvement of STAT3 in the upregulation of pro-angiogenic genes (VEGF and VE-cadherin) (71) and in suppression of miRNA199a transcription (72).

#### STAT3 in MI-Induced Fibrosis and Hypertrophy

In the context of MI-induced fibrosis and hypertrophy, cardiomyocyte-specific ablation of STAT3 exacerbated cardiac fibrosis during the subacute phase of MI, along with an increase in the expression of fibrosis-related genes, most probably due to increased death of cardiomyocytes. Besides, cardiac hypertrophy was enhanced by STAT3 ablation after MI in mouse hearts and consequently, capillary density was perhaps reduced in the border zone (67).

### STAT3 in Pre- and Post-conditioning

Two main signaling pathways have been identified as contributors to the infarct-reducing effect of ischemic preconditioning (73). The first is the Reperfusion Injury Salvage Kinase (RISK) pathway, which involves activation of extracellular regulated kinases ERK1/2 and Akt. The second is the Survivor Activator Factor Enhancement (SAFE) pathway, which involves activation of the JAK-STAT3 signaling cascade. Thus, as a key player of the SAFE pathway, STAT3 mediates cardioprotective effects of ischemic conditioning (74).

The involvement of STAT3 in the cardioprotective effects of conditioning was proved using different animal models, including mice, rats, rabbits, and pigs. It was shown through knocking out STAT3, inhibiting STAT3 with stattic, or inhibiting the JAK-STAT pathway with AG490. Although few studies focused on S727 STAT3 phosphorylation during conditioning, the primary targeted phosphorylation residue is Y705. Starting with the mouse models, Xuan et al. reported that JAK1/2 phosphorylation was increased within 5 min of preconditioning, while Y705 phosphorylation of STAT3 significantly increased within 30 min. Phosphorylation and cardioprotection were blocked by AG490 (75). In addition, cardiomyocyte-specific deletion of STAT3 abolished the cardioprotective effect of both ischemic and pharmacological preconditioning induced by adenosine, diazoxide, and TNF (76). Using the same animal model, it was reported that STAT3 is essential for the cardioprotective effect of ischemic post-conditioning induced by 3 cycles of IR (10 s ischemia-10 s reperfusion). This protocol was shown to induce serine phosphorylation of STAT3 within 10 min of reperfusion. The use of AG490, however, abolished cardioprotection and STAT3 phosphorylation. Surprisingly, modification of the ischemic post-conditioning protocol to 5 cycles of 5 s ischemia-5 s reperfusion showed normal cardioprotection regardless of cardiomyocyte-specific STAT3 deletion. This indicates that cardioprotection by ischemic post-conditioning is dependent on the choice of the post-conditioning protocol, where the number of cycles and duration of ischemia/reperfusion per cycle are a consideration (77).

In rabbit models as well, post-conditioning was shown to induce cardioprotection through the phosphorylation and activation of both JAK and STAT3 proteins, an effect abolished by AG490 (78). In rat models, ischemic preconditioning prior to 30 min of global ischemia and 2 h of reperfusion induced extensive phosphorylation of JAK2 and STAT3 in the pre-conditioned hearts, which was almost completely abolished by an inhibitor of JAK2, AG490 (79). This highlights the importance of JAK2/STAT3 pathway in the cardioprotective effects of pre-conditioning. In the same context, STAT3 has been reported to contribute to the cardioprotective effects of post-conditioning by stimulating respiration and inhibiting MPTP opening (48).

In addition to the *in vivo* animal models, some groups used *in vitro* cellular models of cardiomyocytes and cardiomyoblasts. For instance, Suleman et al. (80) used models of ischemic and pharmacological (i.e., with TNF) preconditioning on WT and STAT3-KO mice-isolated adult mouse cardiomyocytes. Both preconditioning stimuli failed to protect KO cardiomyocytes, and the addition of AG490 abolished preconditioning in WT cardiomyocytes. In a study on H9c2 cells, another team reported that pharmacological post-conditioning with S1P involves JAK-STAT3 activation. The use of AG490 or Stattic abolished the protective effect of S1P, again pointing out the necessity of this pathway in achieving the protective effect (81).

Although a good amount of work on the JAK-STAT3 pathway shows its involvement, solely, in the protective effects of pre- and post-conditioning, some articles have raised a question concerning its role in the RISK pathway. In this context, Goodman et al. claimed that JAK-STAT signaling is insufficient for effective post-conditioning without PI3K-Akt activation in C57bl male mice (82). However, this interlink was not thoroughly investigated. Bearing in mind that SAFE and RISK pathways have common downstream endpoints, and cellular signaling pathways are complexly entangled, the interplay between SAFE and RISK represents an important field of interest for further investigation.

#### Mechanisms of STAT3 Involvement in Ischemic Conditioning

STAT3 participates in conditioning-induced cardioprotection by reducing apoptosis, increasing expression of cardioprotective proteins, protecting mitochondrial respiration, and delaying mPTP opening.

##### Apoptosis

Post-conditioning significantly increases anti-apoptotic Bcl2 expression, along with STAT3 phosphorylation during early and prolonged reperfusion. The use of the JAK inhibitor AG490 abolishes both effects and increases apoptosis, indicating a role of JAK-STAT3 signaling in apoptosis reduction by ischemic post-conditioning (78, 83). In addition, STAT3 was recently reported to be involved in the cardioprotective anti-apoptotic effects of sevoflurane-induced pharmacological post-conditioning (84). Moreover, STAT3 deletion abolished upregulation of anti-apoptotic genes such as induced myeloid leukemia cell differentiation protein (MCl-1), B-cell lymphoma-extra-large protein (Bcl-XL), and cellular FLICE inhibitory protein (c-FLIP) in response to delayed preconditioning (39).

##### Cardioprotective protein upregulation

STAT3 deletion or AG490 administration abolished the upregulation of cardioprotective (COX-2 and HO-1) proteins that were normally expressed in response to delayed preconditioning (39, 85).

##### Mitochondrial STAT3 and ischemic conditioning

Heusch et al. studied mitochondrial STAT3 activation by ischemic post-conditioning. Although previous reports highlighted the role of the S727 residue in translocation of STAT3 to mitochondria, this group reported, using an *in situ* pig model, that ischemic post-conditioning induced an increase in Y705 phosphorylation of STAT3 in the mitochondrial compartment. This was accompanied by protection of mitochondrial respiration and an increase in the calcium retention capacity. Again, AG490 and stattic abolished post-conditioning-induced protection of calcium retention capacity and respiration (44).

In conclusion, STAT3 stands out as an important participant in different types of conditioning protocols. Various studies have reported its phosphorylation and activation following different durations of early reperfusion after conditioning, going up to 120 min. However, no study has determined the kinetics of variation of STAT3 phosphorylation at both, its serine and tyrosine residues, with prolonged durations of reperfusion. Further work focusing on STAT3 kinetics and the interaction with other signaling cascades and kinases would be of insightful.

### STAT3 in Autophagy

Autophagy, a fundamental catabolic process, is involved in lysosomal-mediated degradation and recycling of dysfunctional and unnecessary cellular components (86). This process was shown to be linked to STAT3 in the HL-1 cell line (immortalized mouse atrial cardiomyocytes) and cardiomyocytes (87, 88). In HL-1 cells, STAT3 deficiency lead to increased autophagy, characterized by a significant increase in autophagolysosomes (87). In another study, alcohol was reported to increase autophagy and apoptosis by decreasing STAT3 phosphorylation (88).

**Figure 1:**
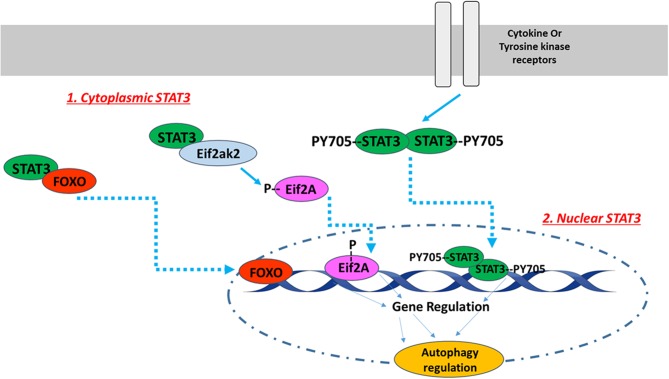
The intracellular localization pattern of STAT3 affects autophagy regulation. The activation of STAT3 downstream of cytokine or tyrosine kinase receptors induces its Y705 phosphorylation and dimerization. STAT3 dimers translocate to the nucleus, where they bind to DNA in order to up- or down-regulate autophagy related genes. In addition to nuclear STAT3, unphosphorylated cytoplasmic STAT3 also regulates autophagy. Unphosphorylated cytoplasmic STAT3 sequesters FOXO and Eif2ak2. FOXO translocates into the nucleus and upregulates multiple autophagy-related genes, while Eif2ak2 phosphorylates Eif2A that regulates autophagy through its nuclear translocation. The image of DNA was adapted from Servier Medical Art (https://smart.servier.com/).

The subcellular STAT3 localization pattern affects autophagy in various ways [Figure 1; (89)]. For instance, nuclear STAT3 can fine-tune autophagy through transcriptional regulation of some autophagy-regulated genes, while cytoplasmic STAT3 inhibits autophagy constitutively through sequestering the eukaryotic translation initiation factor 2 alpha kinase 2 (eif2ak2) and by interacting with other autophagy-related signaling molecules (89). Cytoplasmic STAT3 also interacts with autophagy-related proteins FoxO1 and FoxO3. The latter translocates into the nucleus and upregulates multiple autophagy-related genes. In a recent study conducted with H9c2 cardiomyoblasts, Chen et al. investigated whether angiotensin II-induced cardiomyocyte hypertrophy is affected by STAT3-mediated inhibition of cellular autophagy. They reported that STAT3 modulates autophagy to balance the transcriptional hypertrophic response to angiotensin II stimulation (90). In an attempt to investigate the possible mechanism of this effect, Jonchere et al. showed that STAT3 interacts with an RNA-dependent kinase, PKR, and inhibits phosphorylation of eukaryotic translation-initiation factor 2-alpha (eIF2-α), consequently leading to the reduction of autophagy pathways (91).

Given these specified roles for STAT3 in the regulation of autophagy and noting that autophagy is among the mechanisms of cell death in IR injury (92), an important future approach will be to investigate how STAT3 regulates autophagy during IR injury or cardioprotective ischemic conditioning.

### STAT3 in ER Stress

**Figure 2:**
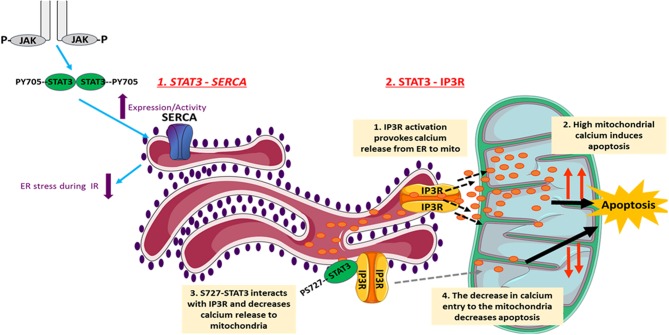
The involvement of STAT3 in the regulation of ER activity. STAT3 decreases cellular death under different conditions through its ER-dependent activity. Following I/R, JAK2-STAT3 pathway rescues SERCA expression and activity, which reduces the ER stress occurring during I/R injury. This reduces cell death and helps in inducing the infarct size. In addition, STAT3 modulates the ER-mitochondrial calcium release by directly interacting with IP3R3 and facilitating its degradation. This consequently leads to a decrease in the calcium release from ER to mitochondria, and thus causes a decrease in apoptotic cell death. The S727 STAT3 residue plays a regulatory role. The images of ER and mitochondria were adapted from Servier Medical Art (https://smart.servier.com/).

The endoplasmic reticulum (ER) plays a major role in cellular calcium homeostasis. Various stimuli such as ischemic insult, oxidative stress, and calcium disturbances lead to the accumulation of unfolded proteins, a condition named ER stress (93). Several studies have investigated involvement of STAT3 in regulating ER activity (Figure 2). However, a recent study exclusively reported unexpected STAT3 localization to the ER, from where it modulated ER-mitochondrial calcium release by directly interacting with IP3R3 and facilitating its degradation in MDA-MB-468 cells. This leads to a decrease in the calcium release from ER to mitochondria, and thus a decrease in apoptotic cell death. The S727 STAT3 residue plays a regulatory role, since a S727 mutation of STAT3 induced excessive release of calcium from ER, followed by H_2_O_2_-induced apoptotic cell death (94). Since the stated mechanism cannot be generalized, its occurrence in heart/cardiomyocytes needs to be investigated.

Regarding ER stress, studies show that it is characterized by impaired activity of sarco/endoplasmic reticulum calcium ATPase (SERCA). On the other hand, well-preserved SERCA activity attenuates ER stress and protects against myocardial IR injury. In this context, using *in vivo* rat model of IR, the JAK2-STAT3 pathway was shown to be involved in rescuing SERCA expression and activity following 3 and 24 h of reperfusion, and thus reducing ER stress and infarct size (95). Similarly, the naturally occurring compound berberine (BBR) protected the rat heart from IR injury through attenuating ER stress via activation of JAK2/STAT3 pathway. Inhibition of the latter blocked attenuation of ER stress by BBR (96). In addition, Banerjee et al. recently proposed that stimulation of mitochondrial STAT3-FAK (focal adhesion kinase) offers a novel therapeutic approach against pathological ER stress in endothelial cells (97). In another *in vitro* study on H9c2 cells, it was shown that the pharmacologic compound Elatoside C protected against hypoxia-reoxygenation-induced ER-stress by activating STAT3 signaling pathways (98).

It seems that the non-canonical activities of STAT3, which were controversially assigned to the maintenance of ETC activity in mitochondria, now include a shift toward activities at the level of ER via IP3R degradation and modulation of calcium flux. Further investigations need to be conducted on cardiomyocytes in this regard, in which it would be interesting to also assess STAT3 in the mitochondrial associated membranes (MAMs) that lie at the interface between mitochondria and ER. This might also help explain why different groups have reported different results regarding the presence of STAT3 in cardiomyocyte mitochondria.

### STAT3 in Cellular Metabolism

**Figure 3:**
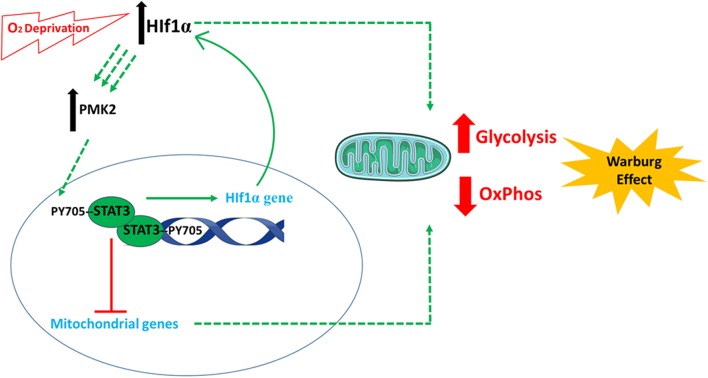
The involvement of STAT3 in cellular metabolism. STAT3 plays an important role in establishing glycolysis-dependent energy derivation, via the Warburg effect. This is mediated by several connections between STAT3 and hypoxia responsive factor 1 alpha (HIF-1α). Following oxygen deprivation (hypoxia), the levels of HIF-1α increase and cause an increase in pyruvate kinase 2 (PKM2) levels. PKM2 induces the phosphorylation of STAT3, which induces the expression of HIF-1α. In parallel, STAT3 also decreases the expression of mitochondrial genes. All these incidences finally favor anaerobic glycolysis and decrease electron transport chain activity and aerobic respiration. The images of DNA and mitochondria were adapted from Servier Medical Art (https://smart.servier.com/).

STAT3 has been considered a potential anti-cancer target since its description as an oncogene in 1999 (41). It is implicated in the definitive metabolic abnormality which characterizes cancer cells: energy derivation using glycolysis (99). Through various activities, STAT3 plays an important role in establishing glycolysis-dependent energy derivation, via the Warburg effect [Figure 3; (100)]. The effects of STAT3 on glycolysis are mainly mediated by several connections between STAT3 and hypoxia responsive factor 1 alpha (HIF-1α). The oxygen sensor HIF-1α is an unstable protein that becomes stabilized under conditions of hypoxia, inducing the activation of glycolysis and the down-regulation of mitochondrial respiration (101). The first connection is that STAT3 transcriptionally upregulates HIF-1α mRNA and protein levels (40), along with attenuating mitochondrial activity by down-regulating mitochondrial proteins in a HIF-1α-independent manner (102). Second, STAT3 is involved in a forward loop which provokes enhancement of aerobic glycolysis and proliferation (103): oxygen deprivation and oncogenes upregulate HIF-1, leading to an increase in pyruvate kinase 2 PKM2 levels. In return, HIF-1 transcriptional activity is enhanced further, and STAT3 is directly phosphorylated by PKM2. STAT3 consequently upregulates HIF-1 expression, which completes the loop (102, 104–108). In a study on MEFs expressing constitutively active STAT3 (STAT3C/C), STAT3C/C increased mRNA for a set of proteins, including PDK-l (pyruvate dehydrogenase kinase 1). This blocked the shuttling of pyruvate into the citric acid cycle in the mitochondria, sending it rather into the glycolytic pathway with resulting increased lactic acid production. Decreased mitochondrial oxidative phosphorylation was also observed in STAT3C/C cells, which was due to the fact that these cells had a lowered amount of mRNA for mitochondrial proteins (ATP synthase, G subunit of the mitochondrial FO complex, fumarate hydratase) (100).

Accumulating evidence indicates that preservation of bioenergetics, including maintenance of fatty acid oxidation, is important for normal cardiac function and prevention of ischemic and non-ischemic heart failure (109–111). The late stage of cardiac hypertrophy is also accompanied by impaired mitochondrial fatty acid oxidation and a shift toward a dependency on glucose metabolism, which is considered inadequate for normal cardiac function (112). To shed light on STAT3's metabolic functions, it is important to briefly reintroduce its involvement in the regulation of mitochondrial functions. As stated previously, STAT3 is known to regulate genes involved in metabolism and mitochondrial activity. Moreover, although its presence is not definitively confirmed in mitochondria of cardiomyocytes of all species, STAT3 is reported to regulate mitochondrial ROS production and respiration in some models. Through these roles, STAT3 is considered a key player in regulating cellular metabolic homeostasis. On the contrary, in a study that focused on the involvement of cardiomyocyte-specific STAT3 in the maintenance of cardiac energy metabolism during angiotensin II-induced hypertension, Altara et al. showed that STAT3 is not essential for the heart to maintain normal fatty acid utilization under stress conditions of cardiac hypertrophy (113).

### STAT3 in Microtubule Stability and Assembly

In cardiac myocytes, microtubules (MTs) are cytoskeleton components that consist of αβ-tubulin heterodimers, assembled into microtubule polymers. Besides their well-established physiological roles, evidence suggests that MTs have an important role in disease conditions, such as cardiac hypertrophy and heart failure. Cooper reported that re-organization of MTs accompanied the development of cardiac disease (114), and an increase in MT numbers correlated with pressure overload-induced pathological cardiac hypertrophy (115, 116). In addition, significant increases in MT number, density, and stability have been reported during heart failure (117).

STAT3 has been reported to partially co-localize with microtubules in various types of cells (118). It contributes to the dynamics and stabilization of MTs by interacting with and inhibiting the activity of stathmin (119, 120), a cytosolic protein that binds αβ-tubulin heterodimers and causes MT depolymerization (42). STAT3 was also reported to physically interact with tubulin, the building block of MTs (121). Nevertheless, the role of STAT3 in regulating MTs of cardiomyocytes is not well-established. In this regard, Ng et al. reported that the gp130 family cytokines (OSM and LIF) induced MT stabilization in neonatal rat ventricular myocytes, and this was inhibited by JAK2/STAT3 inhibitors or STAT3 knockdown (122). Paradoxically, as detected by immunofluorescence, total and tyrosine-phosphorylated STAT3 in cardiomyocytes were located in a pattern that is independent of microtubule distribution in the cells (121, 123). Given the physiological and pathological importance of MT stability in the heart, investigating the role of STAT3 as a regulator of this feature and its connection with physiological and pathological conditions is of great importance.

## Role of STAT3 in Heart Pathologies

STAT3 participates in mechanisms contributing to different chronic cardiac pathologies including hypertension-induced hypertrophy, fibrosis, and diabetic cardiomyopathy (Table 1).

**Table 1:** The roles of STAT3 in various cardiac pathologies.

**Cardiac pathology**	**Roles of STAT3**
Hypertrophy	• **STAT3-dependent hypertrophy** - JAK/STAT3 pathway activated by hypertrophic agonists - IL-6, a main activator of STAT3, induces growth of cardiac cells - Inhibition of STAT3 downregulates collagen synthesis and decreases hypertrophy - Overexpression of STAT3 in CMs increases expression of hypertrophic genes • **STAT3-independent hypertrophy** - Mice with CM-specific STAT3 deletion develop normal pregnancy-induced hypertrophy - IL-6 deletion or chronic LIF treatment does not affect hypertrophy
Fibrosis	• **STAT3 in cardiomyocytes** • STAT3 deletion in CMs induces development of severe fibrosis • STAT3 KO CMs produce paracrine factors that stimulate fibroblast proliferation • STAT3 stimulates induction of pro-fibrotic genes in cardiomyocytes • **STAT3 in fibroblasts** - Activation of STAT3 by AngII stimulates collagen synthesis and fibrosis, while its inhibition induces downregulation of collagen synthesis - STAT3 activation by IL-6 stimulates indirect collagen production in fibroblasts - The cell surface transmembrane ligand EphrinB2 in cardiac fibroblasts has pro-fibrotic actions via synergistic activation of STAT3 and Smad3
Peripartum cardiomyopathy	• Absence of STAT3 leads to PPCM development through oxidative stress
Diabetes	• **Anti-diabetic roles** - Decrease in cardiac STAT3 phosphorylation/activation in type I diabetes - S727 STAT3 phosphorylation decreases streptozotocin-induced type I diabetes - Reduced expression of cardiac STAT3 reported in a type II diabetes model, where downregulation of STAT3 mRNA observed in rats - High glucose induces reduction in Y705-STAT3 phosphorylation and activation in adult rat cardiomyocytes • **Diabetic roles** - Hyperglycemia shown to induce activation of STAT3 signaling pathway through JAK2 - Cardiac levels of p-STAT3 increased in diabetic rats, accompanied by increase in IL-6 and collagen deposition - Increased STAT3 phosphorylation observed in Wistar rats following 21-weeks of high-fat and glucose diet - STAT3 activation reported under high glucose conditions in H9C2 cells and in rat cardiac fibroblasts
Myocardial infarction (Inflammatory approach)	• **STAT3 in cytokine signaling** - STAT3 is a signaling molecule downstream of pro- and anti- inflammatory cytokines: IL-6, IL-1β, TNF-α and IL-10 - Uncontrolled STAT3 activation promotes inflammation: CM-specific mutation of gp130 receptor with uncontrolled STAT3 activation, led to sustained cardiac inflammation in response to MI - Accumulation of non-phosphorylated STAT3 in the nucleus induces expression of pro-inflammatory genes, including IL-6 - STAT3 activation downstream of IL-10 following MI is anti-inflammatory • **STAT3 in neutrophil/macrophage recruitment** - Over activation of STAT3 results in more rapid clearance of neutrophils due to decreased production of the neutrophil-attracting chemokine CXCL1 - Granulocyte colony-stimulating factor (G-CSF), a key mediator of neutrophil infiltration, acts via STAT3 and other signaling pathways - STAT3 demonstrated to be required for expression of Reg3b, which is secreted by cardiac myocytes to facilitate macrophage recruitment • **STAT3 in macrophage polarization** - IL-10-galectin-3 axis essential for M2 macrophage polarization after MI in STAT3-dependent manner - Sodium-glucose cotransporter2 (SGLT2) inhibitors mediate M2c polarization by regulating reactive oxygen and nitrogen species production through STAT3-mediated pathway - STAT3-dependent pathway in post-infarcted rats regulates macrophages phenotype
Doxorubicin cardiotoxicity	- STAT3 overexpression in the heart protects against Dox-induced cardiomyopathy, probably through regulating expression of anti-oxidative MT1 and MT2 - *S*-propargyl-cysteine (SPRC) possesses cardioprotective efficacy against Dox by stimulating STAT3 activation via gp130-mediated signaling *in vitro* and *in vivo*

### STAT3 in Hypertrophy

Cardiac hypertrophy is a compensatory response that allows the heart to cope with the pathogenic stimuli found with many cardiovascular diseases (124). Although it is a compensatory beneficial mechanism, it stands as an independent cause of death by progressing to heart failure. Among the various signaling pathways that mediate cardiac hypertrophy, the JAK/STAT pathway plays a pivotal role in response to different stimuli such as myocardial infarction, cytokines, IR, pressure overload, and neurohormones (124–128). It is activated by hypertrophic agonists [leukemia inhibitory factor (LIF), cardiotrophin-1 (CT-1), and angiotensin II] *in vitro* (124, 128, 129). The main link between STAT3 and hypertrophy induction originated from the fact that the IL-6 family cytokines, which activate JAK-STAT3 pathway, induce growth of cardiac cells.

Several studies highlighted involvement of STAT3 in hypertrophy through its transcriptional roles. Inhibition of STAT3 induced down-regulation of collagen synthesis and regression of hypertrophy, suggesting that STAT3 is a major potential therapeutic target for cardiac hypertrophy (130). In addition, over-expression of STAT3 in mouse cardiomyocytes caused cardiac hypertrophy by increasing expression of hypertrophic genes (ANF, MHC, CT-1) (131).

The inactivation of STAT3 resulting from the loss of gp130 may be a key event in the transition from cardiac hypertrophy to heart failure (58). In this regard, gp130 was reported to play a critical role in hypertrophy through STAT3 activation (132). It was also shown that the hypertrophic growth of neonatal rat cardiomyocytes was induced by LIF through gp130-STAT3 axis. This response was inhibited by overexpressing the suppressor of cytokine signaling negative regulator, SOCS3 (133). Although STAT3 activation has been reported downstream of JAK2 in various cardiac models of hypertrophy, it was demonstrated in an *in vivo* model of hypertrophy that STAT3 becomes phosphorylated with no accompanying JAK2 activation. STAT3 activation contributed to cardiomyocyte growth and survival through its transcriptional activity, and this activation was mediated through the TEC family kinase, BMX, not JAK2 (134).

On the other hand, this prominent role of STAT3 in hypertrophy was questioned by contradicting findings. In this regard, IL-6 deletion or the chronic treatment of C57bl/6J male mice with LIF did not affect development of cardiac hypertrophy (135, 136). In addition, it was reported that mice with cardiomyocyte-specific deletion of STAT3 developed normal pregnancy-induced hypertrophic growth of the maternal heart (137). Overall, the above findings indicate that the role of STAT3 in hypertrophy is still controversial and requires further investigation to establish STAT3-dependent signaling pathways of hypertrophy.

### STAT3 in Fibrosis

Many cardiac diseases and aging are associated with fibrosis in the heart (138). The fibrotic ECM causes increased stiffness and induces pathological signaling within cardiomyocytes, which results in progressive cardiac failure. In addition, excessive ECM impairs the mechano-electrical coupling of cardiomyocytes and increases the risk of arrhythmias (139). Evidence indicates that STAT3 is an important contributor to collagen synthesis and cardiac fibrosis through its activity in fibroblasts and cardiomyocytes.

Under normal physiological conditions, activation of STAT3 is involved in the promotion of cell proliferation and survival, along with the synthesis of ECM in cardiac fibroblasts. The *in vitro* STAT3 activation by IL-6 induced normal production of collagen by fibroblasts (140), and the inhibition of STAT3 by S31-201 induced a pronounced downregulation of collagen synthesis. In this context, Tsai et al. showed that STAT3 activation was stimulated by angiotensin II in rat atrial fibroblasts, indirectly through a paracrine effect linked to collagen synthesis and fibrosis (141). In addition to these studies, increased blood pressure due to ligation of the renal artery in the rat induced cardiac fibrosis, which was attributed to regulation of IL-6 synthesis by cardiac myocytes. Consequently, STAT3 was activated in cardiac fibroblasts and enhanced the expression of collagen (142).

Studying hyperglycemia-promoted fibrosis, Dai et al. reported that STAT3 mediated the proliferation of cardiac fibroblasts and the collagen synthesis by these cells (143). In addition, evidence showed that the cell surface transmembrane ligand EphrinB2 in cardiac fibroblasts has pro-fibrotic actions via the synergistic activation of STAT3 and Smad3, which subsequently become associated (144). Moreover, another study found that the hormone relaxin inhibits TGF-β1-induced cardiac fibrosis by blocking STAT3-dependent autophagy in cardiac fibroblasts (145).

Although the above evidence collectively shows that STAT3 is prominently involved in fibrosis, it was reported that STAT3 deletion in mouse cardiomyocytes induced development of severe fibrosis with advanced age (126). In another study on STAT3-deficient mice, reduced myocardial capillary density was observed within the first 4 months postnatal (57). Evidence showed that STAT3 KO cardiac myocytes produce some paracrine factors that stimulate fibroblasts proliferation.

In addition to the above stated roles, STAT3 is involved in crosstalk between cardiac myocytes and fibroblasts under pathologic conditions. In cardiac cells of mouse hearts subjected to transverse aortic constriction, STAT3 was displaced from the cytoskeletal protein bIV-spectrin at intercalated discs, downstream of Ca^2+^/calmodulin-dependent kinase II (CaMKII) activation. This displacement, induced by CaMKII-mediated phosphorylation of bIV-spectrin, was implicated in cardiac fibrosis and in the loss of cardiac function since it resulted in the nuclear translocation of STAT3 and induction of pro-fibrotic genes in cardiomyocytes (146).

In summary, STAT3 is important for the normal physiologic functioning of fibroblasts and for the ECM synthesis. However, its absence in cardiac myocytes induces fibrosis.

### STAT3 in Peripartum Cardiomyopathy (PPCM)

Peripartum/postpartum cardiomyopathy (PPCM) is a rare heart disease that develops in women during the last month of pregnancy or within 5 months of delivery. Its typical features are apoptosis, inflammation, autoimmune processes, and impaired cardiac microvasculature (147). Observations suggest that cardiac STAT3 is optional during pregnancy, but it is necessary for cardioprotection in postpartum.

The absence of STAT3 leads to PPCM development through oxidative stress. Female mice with cardiomyocyte-specific deletion of STAT3 develop PPCM, associated with the abrogation of superoxide dismutase 2 (MnSOD2) upregulation (137). This consequently leads to an increase in oxidative stress, accompanied by an increase in cathepsin D expression and activity. Cathepsin D provokes formation of a pro-apoptotic and anti-angiogenic cleaved 16 KDa form of prolactin. This in turn stimulates increased miR-146a expression in endothelial cells, which release miR-146a-loaded exosomes that induce subsequent decrease in the metabolic activity of cardiac cells, accompanied by a downregulation of Erbb4, Notch1, and Irk1 expression (148, 149). Moreover, Akt activity is enhanced in PPCM downstream of prolactin, which increases the redox imbalance and SOD2 loss due to down-regulation of the anti-oxidative transcription factor FoxO3A (149).

Low levels of ventricular STAT3 contribute to a state of energy depletion and increased ROS generation, which compromises glucose uptake and increases the sensitivity of the heart to the toxic effects of chronic β-adrenergic signaling. This again assures the importance of STAT3 in cardioprotection against PPCM (150). Generally, cardiac glucose uptake and oxidation are decreased upon chronic stimulation with the β-adrenergic agonist isoproterenol. Cardiac myocytes mostly rely on glucose oxidation under these conditions, since isoproterenol depletes serum free fatty acids, and cardiac free fatty acids uptake. Evidence showed that inadequate glucose uptake by cardiac myocytes due to STAT3 loss was associated with increased mitochondrial ROS formation caused by insufficient NADPH generation needed to maintain adequate GSH recycling in mitochondria (2).

### STAT3 in Diabetic Cardiomyopathy

Diabetes mellitus (DM) is one of the most prevalent diseases worldwide (151). Diabetic patients have a higher chance of developing cardiovascular complications due to the fact that diabetes exerts harmful effects on the cardiovascular system (152, 153). Diabetic cardiomyopathy (DCM) is defined by the presence of abnormal myocardial structure and performance in the absence of other cardiac risk factors in individuals with diabetes mellitus (154). Therefore, it is an independent complication of diabetes characterized by myocardial dysfunction. DCM is characterized by diastolic dysfunction caused by myocardial fibrosis which occurs in response to hyperglycemia (155).

Under non-ischemic cardiac conditions, the effect of diabetes on the expression, phosphorylation, and activation of cardiac STAT3 is controversial. On one hand, various papers have reported a decrease in cardiac STAT3 phosphorylation and activation in several diabetic experimental models. In this regard, Wang et al. showed that myocardial pS727-STAT3 activation was decreased in streptozotocin-induced type I diabetes (156). Using the same model, the same team later demonstrated that pY705-STAT3 phosphorylation was also decreased (157, 158). In addition to the diabetes I model, reduced expression of cardiac STAT3 has been reported in a type II diabetes model, where a downregulation of STAT3 mRNA was observed in rats (159). Along the same lines, exposing isolated adult rat cardiomyocytes to high glucose conditions induced a reduction in Y705-STAT3 phosphorylation and activation (160). Similarly, both Y705 and S727 STAT3 phosphorylation levels were significantly decreased in H9c2 cells (rat cardiac myoblasts) subjected to high glucose conditions (157, 161).

On the other hand, other studies have reported that STAT3 phosphorylation was significantly increased in diabetic hearts. Hyperglycemia was shown to induce activation of STAT3 signaling pathway through JAK2 (162, 163). In diabetic rats, cardiac levels of p-JAK2 and p-STAT3 are significantly increased, accompanied to a significant increase in IL-6 and collagen deposition (162). In the same context, streptozotocin-induced diabetes resulted in cardiac STAT3 activation in C57BL/6 mice (164). Similar patterns were observed in a rat model of streptozotocin-induced diabetes (165). In agreement with these studies, an increased level of STAT3 phosphorylation was observed in Wistar rats following 21-weeks of high-fat and glucose diet (166). In addition to *in vivo* studies, STAT3 activation was also reported under high glucose conditions in H9c2 cells (165, 167) and rat cardiac fibroblasts (143).

As previously discussed, STAT3 is known to be involved in the mechanisms of fibrosis, which is a prominent feature of DCM. Thus, studying involvement of STAT3 in myocardial fibrosis specifically during diabetes presents a particular prominent interest for understanding DCM. In this context, cardiac fibrosis was recently shown to be associated with increased matrix metalloproteinase-9 (MMP-9) expression in DCM (168). In addition, connective tissue growth factor (CTGF) expression was observed to be increased in different fibrotic disorders, mediating DCM development (169). As a transcription factor, STAT3 has been reported to regulate MMP9 and CTGF expression in diabetic rats with cardiac fibrosis, and suppression of STAT3 inhibited fibrosis (170).

Diabetes constitutes a prominent risk factor in the development of ischemic heart disease. Thus, the effect of DM on the sensitivity of the heart to IR injury is of major interest. Clinical studies have revealed that DM worsened long-term outcome of ischemic heart disease by increasing sensitivity to IR injury, but the acute outcome following IR injury remains controversial (153). In this regard, the infarct size was reported to be significantly increased, unchanged, or decreased in diabetic rats compared to controls (156, 157, 171). Despite the contradictions in the effect on infarct size, the post-ischemic activation of cardiac STAT3 was reported to be reduced in all the investigations (153). In rat hearts subjected to IR, a reduction in pY705-STAT3 was observed in diabetic rats (172). In other *in vivo* studies, the same reduction was reported in different models (156, 157, 160, 173). With regard *in vitro* models, STAT3 inactivation was reported in cultured rat myocytes exposed to high glucose and hypoxia/reoxygenation (156). In addition, in cultured H9c2 cells exposed to high glucose and subjected to hypoxia/reoxygenation, pS727 STAT3 and pY705 STAT3 levels were decreased following ischemic injury (160).

In conclusion, the decrease in STAT3 phosphorylation/activation in post-ischemic hearts due to diabetes represents a definitive response regardless of the model. This might consequently lead to increased sensitivity to myocardial IR injury in diabetes. Thus, STAT3 might be considered in future therapies targeting the adverse effects of diabetic cardiomyopathy.

### STAT3 in Myocardial Infarction: Focus on Inflammation

Myocardial infarction represents the most common form of acute cardiac injury. Among the variable mechanisms underlying myocardial IR injury, inflammation constitutes the hallmark of MI and reperfusion injury (174). The post-MI inflammatory response includes two phases. The first phase is characterized by the recruitment and infiltration of inflammatory cells to the infarcted area. The second phase comprises the resolution of inflammation (175). STAT3 plays roles in regulating cardiac remodeling following MI, partly through its involvement in the inflammatory response underlying this phenomenon (67). In this regard, STAT3 functions by interfering with the production of key inflammatory cytokines following MI (1), in the infiltration of neutrophils and monocytes to infarcted area (176), and in the regulation of macrophage polarization (177, 178).

#### STAT3 in Inflammatory Cytokine Signaling

STAT3 is a central signaling molecule for many cytokine receptors, including pro-inflammatory and anti-inflammatory ones. Thus, it participates in both inflammatory and non-inflammatory responses downstream of cytokine activation. Following MI, and as a part of the pro-inflammatory response, an increased secretion of pro-inflammatory cytokines such as IL-6, IL-1β, and TNF-α is observed (1).This increased secretion could be detrimental if it exceeds the acute phase. As already stated, STAT3 is acutely activated downstream of IL-6 *via* the gp-130 receptor, and this activation is regulated by subsequent inhibition by SOCS3 (179). In the context of inflammation following MI, a cardiomyocyte-specific mutation of the gp130 receptor in a mouse model lead to uncontrolled STAT3 activation, expectedly due to the interruption of the SOCS3 negative feedback loop (180). This was accompanied by a sustained cardiac inflammation in response to MI. Therefore, uncontrolled and continued gp130-STAT3 activation promotes cardiac inflammation following MI, suggesting a pathogenic role for high IL-6 levels and inflammation as a result of activated signaling pathways in MI (176).

On the gene expression level, evidence showed that the accumulation of non-phosphorylated STAT3 in the nucleus can induce the expression of pro-inflammatory genes, including IL-6 (181).

On the other hand, and as part of the anti-inflammatory response, STAT3 is activated by IL-10. STAT3 activity by itself does not result in an anti-immune response. Instead, STAT3 activates expression effector genes that selectively repress sets of inflammatory genes such as TNFα, IL-12p40, IL-1α, IL-1β, IL-6 (182, 183). In short, later STAT3 activation, linked to IL-10, is beneficial and anti-inflammatory; however, initial brief STAT3 activation, linked to IL-6, is pro-inflammatory and harmful if too robust.

#### STAT3 in Neutrophil/Macrophage Recruitment

IL-6 represents an important checkpoint regulator of neutrophil trafficking during the inflammatory response by orchestrating chemokine production and leukocyte apoptosis. In a study that aimed to understand the receptor-mediated signaling events responsible for IL-6-driven neutrophil trafficking, Fielding et al. demonstrated that hyper-activation of STAT3 in mice with a global gp130 knock-in mutation that does not bind SOCS3 resulted in a more rapid clearance of neutrophils, and this coincided with decreased production of the neutrophil-attracting chemokine CXCL1. Thus, the IL-6-driven signaling via STAT3 controls the clearance of inflammatory neutrophils (176). The apparent discrepancy between cardiac-targeted (discussed above) vs. global gp130-mediated STAT3 hyperactivation in sustaining or restraining an inflammatory response highlights the need to better understand the role of STAT3 activation in a cell-specific and context-dependent manner.

Granulocyte colony-stimulating factor (G-CSF), a key mediator of neutrophil infiltration, is significantly increased and can be induced by angiotensin II. In a mouse study, angiotensin II infusion lead to a significant increase in G-CSF, along with a neutrophil accumulation, pro-inflammatory cytokine expression, reactive oxygen species production and cardiac fibrosis via activation of STAT3, ERK1/2, and AKT signaling pathways (126).

STAT3 plays a role in the recruitment of macrophages. This process is facilitated by Reg3b secretion from cardiac myocytes in response to the gp130-family cytokine oncostatin M (OSM), and STAT3 was demonstrated to be required for the expression of Reg3b (184).

#### STAT3 in Macrophage Polarization

Macrophages are major effector cells in tissue repair and fibrosis. They can be broadly categorized as M1 and M2. M1 macrophages are pro-inflammatory, expressing inflammatory genes, including iNOS. In contrast, M2 macrophages are involved in the resolution of inflammation and secrete arginase-1 and IL-10 (177). Accumulating evidence shows that STAT3 plays key roles in determining the polarization of macrophages to the M2 phenotype (178).

In this context, the IL-10-galectin-3 axis was recently shown to be essential for the M2 macrophage polarization after myocardial infarction in a STAT3-dependent manner, and these macrophages contributed to tissue repair by promoting fibrosis and clearance of apoptotic cells (185). In another recent study, sodium-glucose cotransporter2 (SGLT2) inhibitors mediated M2c polarization by regulating reactive oxygen and nitrogen species production through a STAT3-mediated pathway (186). Similarly, Lee et al. demonstrated that a STAT3-dependent pathway in post-infarcted rats regulates macrophages phenotype (177).

Overall, STAT3 plays pivotal roles in regulating the cardiac response during myocardial infarction, partly through regulating inflammation. It interferes in the pro-inflammatory and anti-inflammatory responses, whereby STAT3 is first activated downstream of inflammatory cytokines and then regulates the infiltration of immune cells to the injured area.

### STAT3 in Doxorubicin-Mediated Cardiotoxicity

Doxorubicin (Dox) is an important anticancer agents. However, its clinical application is limited by severe cardiotoxicity (187). Various mechanisms are suggested to be involved in Dox-induced heart failure. They include DNA intercalation, free radical generation, and damage to cell membranes. Many studies support the notion that oxidative stress plays a significant role in the pathogenesis of Dox-induced cardiotoxicity (188). STAT3 has been shown to be involved in cardioprotection against Dox, whereby the overexpression of STAT3 in the heart was shown to be protective against Dox-induced cardiomyopathy (131). One proposed mechanism is through regulating the expression of the anti-oxidative proteins metallothionein 1 and 2 (MT1 and MT2); the JAK2/STAT3 pathway was reported to be partially involved in the MT-mediated protection against Dox-induced cardiotoxicity (187). In addition, another study showed that *S*-propargyl-cysteine (SPRC), a source of endogenous hydrogen sulfide (H_2_S), possesses cardioprotective efficacy against Dox by stimulating the activation of STAT3 via gp130-mediated signaling *in vitro* and *in vivo*. In Dox-stimulated cardiotoxicity, SPRC enhanced cell viability, restored expression of gp130/STAT3-regulated downstream genes, inhibited apoptosis and oxidative stress, and antagonized mitochondrial dysfunction. All of these beneficial effects of SPRC were lost by blocking the gp130/STAT3 signaling (33).

## Conclusion and Perspectives

STAT3 represents a crucial molecule for the maintenance of heart function, under physiological and pathological conditions. The pathological conditions in which STAT3 is involved include myocardial infarction, diabetic cardiomyopathy, peripartum cardiomyopathy, fibrosis, and hypertrophy. This involvement of STAT3 is mainly mediated through its functioning as a transcription factor able to regulate a wide array of genes. However, several functions are still controversial and need to be better understood in order to guide future therapeutic strategies against these pathologies. The roles played by STAT3 in autophagy, metabolism, and ER stress need to be better established in the context of the mentioned cardiac pathologies in order to link the abnormalities at the molecular and functional levels (189, 190). Moreover, the interplay between STAT3 and other signaling molecules needs to be deciphered for a better understanding of the regulators of STAT3 functions. In addition, the pattern of STAT3 distribution between mitochondria, ER and MAMs needs to be investigated under physiological and pathological conditions. In the context of inflammation, the involvement of STAT3 in regulating the inflammatory response needs to be studied under protective conditions, not only pathologic conditions, in order to investigate if STAT3 induces cardioprotection by regulating inflammation.

The level of STAT3 activation in the heart likely determines whether its actions are beneficial, harmful, or a combination of the two. For instance, transgenic mice with cardiac-specific overexpression of a constitutively active STAT3, which produces low levels of continuous activation, demonstrated evidence for VEGF induction in the heart (71).On the other hand, while protecting against DOX-induced cardiomyopathy via the induction of protective factors, cardiac-specific overexpression of STAT3 resulted in clear evidence for pathological hypertrophy (131). Additionally, co-factors and post-translational modifications, as well as its subcellular localization (e.g., mitochondria), are likely important in determining the flavor of increased STAT3 activation.

Therapeutically, understanding the mechanisms underlying a pathology represents the first step toward discovering preventive or treatment protocols and medications against this pathology. Since STAT3 is involved in a wide range of physiological and pathological cardiac conditions, understanding the mechanisms of this involvement paves the way toward finding therapeutic strategies involving either the inhibition or activation of STAT3. By describing the different roles of STAT3 as a transcription factor and a signaling molecule, and through explaining its upstream activation, the current review highlights several targets for therapeutic approaches. This may include pharmacological activation or inhibition of STAT3, respectively achieved by inducing the secretion of upstream activators (such as cytokines or growth factors) or developing direct pharmacological inhibitors with non-toxic side effects.

## Author Contributions

All authors have equally contributed to the writing of the manuscript and have approved the final version for submission.

### Conflict of Interest

The authors declare that the research was conducted in the absence of any commercial or financial relationships that could be construed as a potential conflict of interest.
